# Nanobodies in the fight against infectious diseases: repurposing nature's tiny weapons

**DOI:** 10.1007/s11274-024-03990-4

**Published:** 2024-05-21

**Authors:** Soha S. Rizk, Dina M. Moustafa, Shahira A. ElBanna, Hanzada T. Nour El-Din, Ahmed S. Attia

**Affiliations:** 1 Microbiology and Immunology Postgraduate Program, Faculty of Pharmacy, Cairo University, Cairo, 11562 Egypt; 2 Department of Medical Sciences, Faculty of Dentistry, The British University in Egypt, El Sherouk City, Cairo, 11837 Egypt; 3 Department of Microbiology and Immunology, Faculty of Pharmacy, Cairo University, Cairo, 11562 Egypt

**Keywords:** Bacteria, Diagnosis, Infectious diseases, Nanobodies, Prophylaxis, Treatment, Viruses

## Abstract

Nanobodies are the smallest known antigen-binding molecules to date. Their small size, good tissue penetration, high stability and solubility, ease of expression, refolding ability, and negligible immunogenicity in the human body have granted them excellence over conventional antibodies. Those exceptional attributes of nanobodies make them promising candidates for various applications in biotechnology, medicine, protein engineering, structural biology, food, and agriculture. This review presents an overview of their structure, development methods, advantages, possible challenges, and applications with special emphasis on infectious diseases-related ones. A showcase of how nanobodies can be harnessed for applications including neutralization of viruses and combating antibiotic-resistant bacteria is detailed. Overall, the impact of nanobodies in vaccine design, rapid diagnostics, and targeted therapies, besides exploring their role in deciphering microbial structures and virulence mechanisms are highlighted. Indeed, nanobodies are reshaping the future of infectious disease prevention and treatment.

## Introduction

The discovery of monoclonal antibodies (mAbs) has significantly influenced the field of biological industries. This was implemented by Orthoclone, the first Food and Drug Administration (FDA) approved mAb which has a crucial role in preventing rejection in organ transplantation (Starzl and Fung 1986). Since then, an enormous number of mAbs have been well-established and marketed for their beneficial clinical applications including targeted treatment and enhanced therapeutic precision. However, mAbs use was restricted owing to the sophisticated structure and large size which affect their binding specificity, tissue penetration, and clearance time in certain diseases (Buss et al. 2012). Additionally, the synthesis and production of mAbs are costly and time-consuming.

Coincidence plays a vital role in most of the scientific breakthroughs and the same narrative transpired with the first observation of a peculiar antibody molecule that later became a defining milestone in history, currently known as nanobodies (Nbs). In 1993, the Hamers’ lab serendipitously discovered naturally occurring heavy-chain antibodies in the serum of the camel (Hamers-Casterman et al. 1993). Later in 1995, Greenberg and co-workers detected single-domain antibodies from nurse sharks (*Ginglymostoma cirratum*) (Greenberg et al. 1995). These molecules differ from their conventional ones in their composition which includes only the heavy-chain variable dimers while missing their light-chain counterparts. Nevertheless, they possess an extensive antigen-binding repertoire.

Nanobodies (Nbs) “also referred to as single-domain antibodies (sdAb)’’ are the antigen-binding molecules engineered from the camelid or sharks heavy chain antigen-binding domain that are called the camelid variable heavy-chain region (VHH) and immunoglobulin new antigen receptor (VNAR), respectively (Schrankel et al. 2019). While the human IgG immunoglobulin weighs ~ 150 kDa, the heavy-chain antibody weighs ~ 80 to 90 kDa, and the derived Nbs are ~ 12 to 15 kDa (Pillay and Muyldermans 2021; Vincke and Muyldermans 2012). They are one-tenth the size of a normal antibody (Schrankel et al. 2019), making their production and utilization far more applicable. They also possess low immunogenicity owing to their small size, which is around 110 amino acids (~ 4.4 nm high; ~ 2.5 to 2.8 nm diameter) (Cortez-Retamozo et al. 2004; Sánchez-García et al. 2021). Moreover, Nbs can bind to embedded epitopes that are not accessible to complete antibodies and have a greater affinity and selectivity in targeting the active sites of enzymes and receptors. It is worth noting that Nbs exhibit remarkable stability, demonstrated by their ability to withstand some drastic conditions of pH, pressure, and temperature while maintaining their antigen-binding capacity. They can tolerate extreme pHs (pH 3.0–9.0), and high pressure (500–750 MPa) (Jovčevska and Muyldermans 2020). Nbs are also known to exhibit long shelf-life with high storage stability at different temperatures; 4 °C and -20 °C for long storage periods (months), and 37°C for shorter ones (weeks). Moreover, some studies reported their heat tolerance to higher temperatures (60–80 °C). Yet improper Nbs refolding by heat denaturation represents a great concern. Furthermore, Nbs demonstrate high stability against proteolytic enzymes and some chemical denaturants like urea (De Vos et al. 2013; Jovčevska and Muyldermans 2020). On another front, the specificity of the Nb can be generated from cell-based microbial expression systems such as *Escherichia coli*, yeasts, or cell-free platforms (using ribosomes) (Schrankel et al. 2019). This simple yet critical approach can have a significant effect on the reduction of Nbs production costs.

Nbs have already been used in diverse fields and particularly notable is the first Nb approved for a therapeutic indication in 2018, named Caplacizumab, which is used for acquired thrombotic thrombocytopenic purpura (Duggan 2018). Nbs are tested in a wide range of prospective innovations, such as investigating the viability of the VHHs in phage display, testing its potential in shampoos for dandruff reduction and introducing the first evidence of Nbs inhibiting the cell-free and cell-to-cell transmission in hepatitis C infection (Dolk et al. 2005; Tarr et al. 2013). In addition, Nbs are also tested to serve in identifying tumor cells by targeting human growth factor cell receptors (HER2) and carbonic anhydrase IX (CAIX) (Keyaerts et al. 2016; Kijanka et al. 2016). The wide array of possible revolutionary applications offered by these small biomolecules will inevitably boost Nbs utilization. In the current review, the Nbs’ structure, methods of production, advantages, disadvantages and potential applications will be discussed with emphasis on their potential role in infectious diseases.

### The structure of different forms of antibodies

**Fig. 1:**
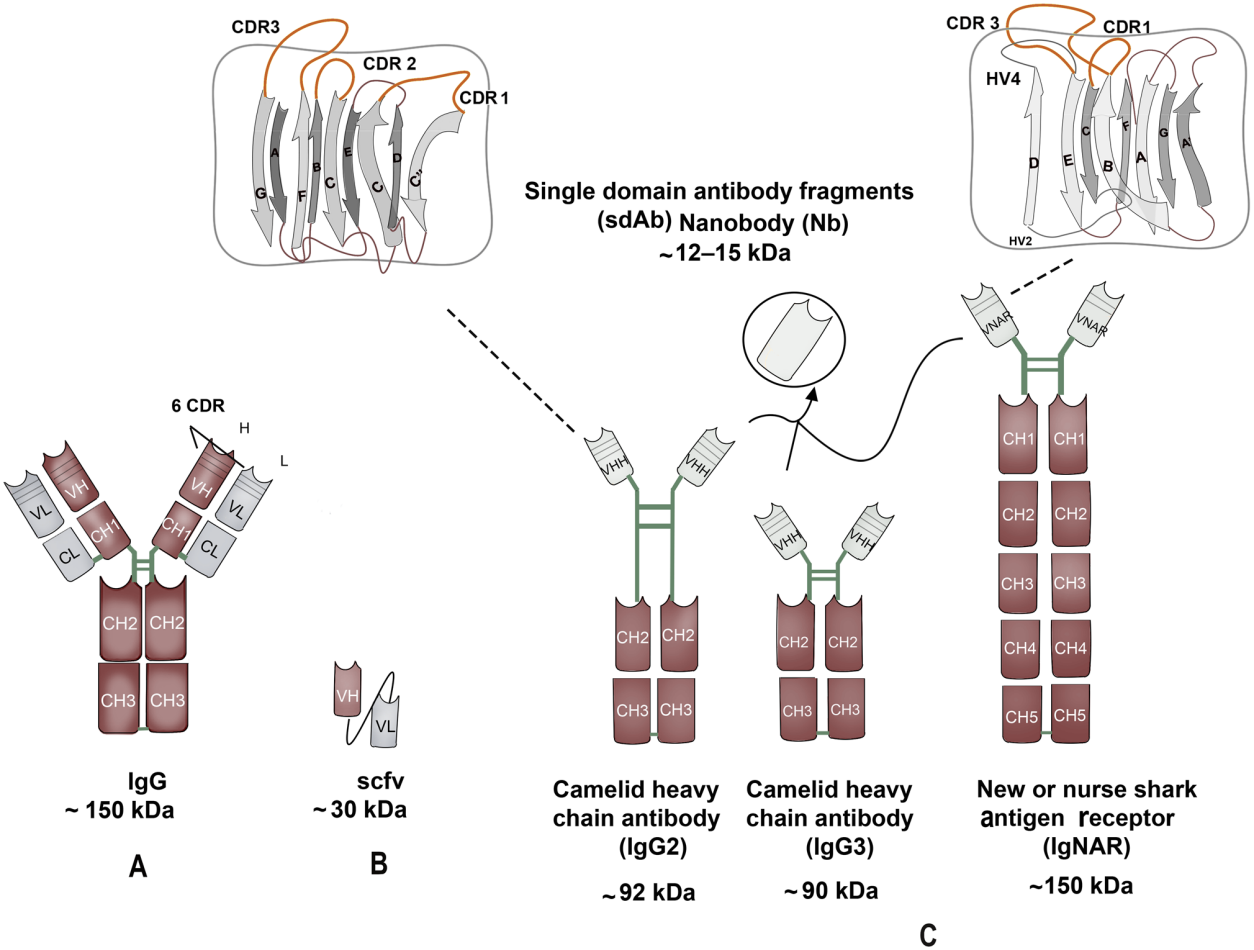
Comparison of Nbs’ structure to other antigen-binding moieties. A schematic diagram showing the difference between Nbs and other antigen-binding moieties. **A** Conventional antibody with its heavy chain (VH) (pink color) and light chain (VL) (gray color). **B** Single chain fragment variable (scFv), which contains a pair of VH and VL domains connected by an oligopeptide bond. **C** Three different heavy chain antibodies; the camelid heavy chains contain VHH segment, hinge, CH2 and CH3 with long (IgG2) or short hinge (IgG3), and the shark heavy chain containing one variable domain and five constant domains. The three heavy chains exhibit single domain antibody (sdAb). The sdAb is formed of three hypervariable sections surrounded by nine β-sheet strands connected by disulfide bond

To properly understand the distinctiveness of Nbs, a structural comparative overview of Nbs, conventional antibodies, and the parent heavy chain antibodies is presented (Fig. 1) and discussed as follows.

The binding specificity of the full-length antibodies is determined by variable regions in their heavy (VH) and light chains (VL). The two light chains are composed of a variable domain (VL) and a constant domain (CL). The VH, CH1, hinge, CH2, and CH3 domains make up the two heavy chains (VH), with the CH1 domain serving as a key connection between the heavy and light chains (Muyldermans 2013; Wanner et al. 2021). Collectively, they generate a diversity of at least 10^15^ B-cell receptors (BCRs) in humans (Mitchell and Colwell 2018). The linkage of the CH2 and the CH3 makes the crystallizable fragment (Fc) portion of the antibody while the antigen-binding (Fab) region is composed of the heavy chain's outer domains (CH1 & VH) as well as the light chain's variable and constant domains (CL & VL). The pairing of the VH-VL by an oligopeptide generates the smallest functional antigen-binding unit, known as the single-chain fragment variable (scFv), with a size of ~ 30 kDa that can be created from the full-size antibodies (Muyldermans 2013). However, unlike VHHs, scFvs have lower affinities, reduced half-life, and stability, as well as lower thermostability when compared to their parent antibodies. As a result, there is a higher probability of aggregation and subsequent risk of immunogenicity (Bates and Power 2019).

The camelid heavy-chain antibodies on the other hand lack both the light chains and the CH1, which gives them an advantageous small size, with a molecular weight of ~ 90 kDa. The dromedary heavy-chain antibodies carry only the VHH segment, hinge, CH2 and CH3 fragments with a direct connection of the rearranged VHH exon to the hinge region belonging to one of two types of hinge isotypes: long (IgG2) or short (IgG3), referring to the fraction's hinge length. In this case, antigen recognition is through the variable domain of the heavy chain. Accordingly, the compact design of Nbs allows better adaptability for hidden targets (Arbabi-Ghahroudi 2017; Muyldermans 2013). Similarly, the antibodies devoid of light chains found in cartilaginous fish consist of one variable domain followed by five constant domains [(V-NAR)-5(C-NAR)] (Deffar et al. 2009; Zielonka et al. 2015).

The VHHs molecules derived from the camelid heavy chains restrict the antigen binding to a single domain of about 110 amino acids. These molecules comprise three hypervariable sections (HV) that localize the sequence variation of the variable domains (V) and are surrounded by a conserved framework (FR). Nine β-sheet strands (A-B-C–C'-C'-D-D-E–F-G) make up the folded variable domain, which is arranged into four-stranded β-sheets and five-stranded β-sheets joined by loops and a conserved disulfide bond. The HV regions are arranged into three loops (H1, H2, and H3) that connect the stranded β-sheets. A continuous surface is formed by the cluster at the N-terminal that is complementary to the surface of the epitopes or antigens (paratope) and this area is referred to as the complementarity-determining region (CDR). The sequence within the loops is highly variable, but the extent of the variation is limited except for the H3 loop (CDR3) (Muyldermans 2013). Controversially, the conventional antibodies were thought to have wider diversity compared to Nbs as the latter have paratopes of smaller size. However, this notion was disproven by the large H1 loop (CDR1) that is responsible for antigen recognition and was found to be longer than those in the variable domain of the conventional antibody’s heavy chain, subsequently serving in largening the paratope size and exhibiting diverse loop architectures (Nguyen et al. 2000). Within the conserved FR2, the highly conserved hydrophobic amino acids normally found in the full-size antibodies, are replaced in VHHs with more hydrophilic amino acids, rendering them more soluble (Asaadi et al. 2021; Muyldermans 2013).

The VNAR domains, on the other hand, are members of the immunoglobulin’s superfamily and hence they have a β-sandwich structure. The VNARs lack the hinge region yet there is a wide space for interacting with multiple epitopes which is enhanced by the dimerization between C1 and C3 domains. Unlike the mammalian variable domain counterpart, the β-sandwich fold in the VNAR only has eight strands instead of ten. With a size of roughly 11–12 kDa, the VNARs are believed to be the smallest antibody-like antigen-binding domains known in the animal kingdom (Stanfield et al. 2004; Zielonka et al. 2015). This structure results in fewer antigen-binding loops (CDR1 & CDR3) compared to antibodies, but the elongated CDR3 compensates for this (Feige et al. 2014; Könning et al. 2017; Zielonka et al. 2015). Still, the VNARs' diversity, like that of the VHHs, is predominantly seen in the CDR3 sequences. Two cysteines in FR1 and FR3 form a stabilizing disulfide bond, and additional ones in CDR3 can provide extra stability (English et al. 2020; Feige et al. 2014; Feng et al. 2019).

In conclusion, compared to the standard antibody binding sites, antigen-binding sites in VHHs and the VNARs are smaller in terms of molecular surface area and diameters. They differ from the typical canonical structures of the full-length antibody in their non-canonical CDR1 and CDR2 structures, as well as an elongated CDR3 loop length distribution. However, they have similar amino acid compositions and as a group they appear to be no longer in the distance measured from the CDR base to the tip than the conventional antibodies (Henry and MacKenzie 2018). For protein-binding, rather than operating six-loop configurations like typical antibodies, Nbs only use their three CDR loops. They exert their expanded CDR3 loop to penetrate the active site or the CDR2 loop in circumstances where the Nb's standard CDR3 loop is insufficient to protrude to the antigen. (Desmyter et al. 2002; Henry and MacKenzie 2018; Sela-Culang et al. 2013).

## Production of nanobodies

**Fig. 2:**
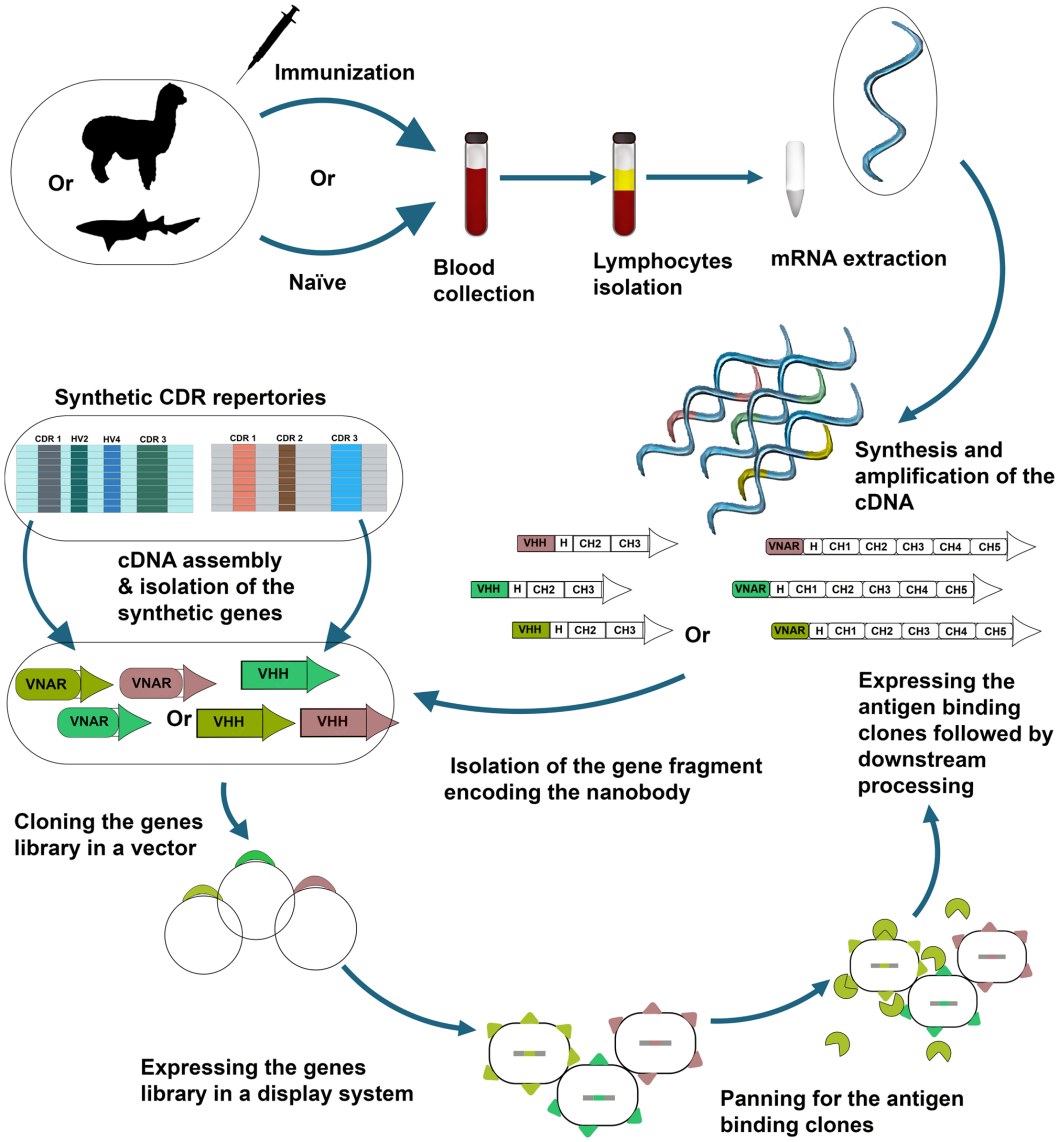
Nanobodies generation process. A schematic diagram for the different generation approaches of Nbs including immune, naïve, and synthetic libraries. The diagram is partially generated using BioRender

The production of sdAb fragments traditionally entails the amplification of VHH or VNAR gene segments at an affordable low cost. They are then cloned into a display system, whether it is a bacteria, yeast, phage, or ribosome, followed by the generation of a large collection of clones "library" accompanied by biopanning of the high-affinity antigen-specific clones and their retrieval (Fig. 2).

Inherently, for immune library generation, the stages of Nbs production generally commence by immunizing healthy young adults including dromedaries, camels, llamas, alpacas, or sharks with a protein cocktail to generate a library of at least 10^6^–10^8^ individual clones (Müller et al. 2012; Muyldermans 2021b). Over the course of a few months, the animals can be routinely injected with the target immunogen. Since the used animals are outbred, it is recommended that more than one is immunized at this early stage. Each animal is thought to elicit a different immune response, with a subsequent large repertoire of Nbs from which the best-performing clone is selected (Muyldermans 2021b). Affinity maturation and class switch recombination are induced by deliberate repeated immunization which leads to boosting the odds of detecting VHHs with the targeted functional features that may not be existent in naïve libraries (Ingram et al. 2018). Extraction of mRNA is done from the blood acquired after the immunization step, then the mRNA is transformed into cDNA and utilized to amplify the VHH gene segments (Muyldermans 2021b). Constructing libraries through animal immunization has some limitations such as being time-consuming and costly, and it may also generate redundant subpopulations of certain antigens. Additionally, when it comes to non-immunogenic molecules like RNA or DNA, which fail to elicit an immune response, they are not the best choice (Muyldermans 2021b; Sabir et al. 2014).

Another significant limitation of the immunization libraries is the limited target space for sensitive proteins. Targets, such as many human membrane transporters, easily unfold upon injection primarily due to the adjuvants used and the dromedary's high body temperature. Additionally, unless their affinities are extremely high, non-covalent ligands dissociate from the protein shortly after injection, making it difficult to promote target conformations (Zimmermann et al. 2018). It is worth noting that immunization requires access to animal facilities, and this may not always be feasible. On another front, and from an ethical point of view, animal usage to that end is strongly discouraged for compounds that are poisonous, contagious, or harmful to both animals and environment. Hence, steering directions are currently implemented towards the use of other Nb repertoires such as naïve and synthetic libraries which do not require animals to be immunized against bacteria, viruses, or toxoids (Gray et al. 2016).

As for the construction of a naïve library, a pool of blood from multiple non-immunized animals is required. This method has the advantage of being rapid and the ability to recover a VHH repertoire that should at least acquire the size of 10^9^–10^11^ clones, with the added benefit of being more diversified (Muyldermans 2021b; Sabir et al. 2014). On the other hand, taking up to 10 L of blood to build a diversified naïve Nb library with around 10^10^ different VHH clones is tedious (Muyldermans 2021b). However, the procedure has been found to yield over-adequate Nb libraries of a size of 10^7^ with as little as 23 mL, from which high-affinity Nbs can be extracted (Sabir et al. 2014).

Synthetic libraries are the third source for Nbs and they can provide access to bigger repertoires without the benefits of target immunization and affinity maturation (Ingram et al. 2018). A stable and well-expressed Nb scaffold, preferably with a crystal structure, is usually chosen for the construction of a synthetic library without the need to draw blood from animals (Muyldermans 2021b). Synthetic libraries have a diverse clone size of 10^9^–10^15^ and often exhibit a single shape and are randomized in only one region of their surface (Muyldermans 2021b; Zimmermann et al. 2018). A single or a few Nbs with desirable biochemical features are randomly selected and their sequences are amplified by PCR (Muyldermans 2021b). Following that, the PCR products are ligated into phage or ribosome display, or both and three synthetic Nbs selection platforms tailored to membrane protein targets are then engineered with varying CDR3 loop lengths and configurations. The Nb library is displayed using both phage and ribosome systems and created by analyzing many deposited camelids VHHs structures (Zimmermann et al. 2018).

It is also worth noting that Nb libraries can be developed from human origins through phage-display technology under the hypothesis that certain VH framework sites can compensate for the loss of the light chain, resulting in soluble human Nbs (Wu et al. 2020). A previous study reported cloning of 17 human germline immunoglobulin heavy chain variable region (IGHV) alleles, and expressing them in *E. coli*, and then characterizing their properties, along with a camelid Nb as a control. Another previously experimented approach is that fully human single-domain antibodies were obtained by grafting the complementarity determining regions (CDR1, CDR2, and CDR3) from naïve libraries into the FR regions of a human germline immunoglobulin VH variable region allele (Wu et al. 2020). This technology promises antibodies derived entirely from human sequences which exhibit less immunogenicity compared to camelid or humanized Nbs, leading to improved safety and efficacy for human use.

Nbs can be expressed in both prokaryotic and eukaryotic systems, such as *E. coli*, *S. cerevisiae*, and *Pichia pastoris*. The most common approach for generating Nbs is to promote their secretion in the *P. pastoris* or the *E. coli* periplasm (Chen et al. 2019; de Marco 2020). The periplasm's oxidizing conditions promote the formation of disulfide bonds, which help in stabilizing the Nb structure. After an osmotic shock step to permeabilize the bacterial outer membrane, the folded binders are normally recovered in the supernatant and affinity purification is used to recover the Nbs (de Marco 2020). It is also noted that upon precipitation of the highly temperature-sensitive *E. coli* proteins, heat incubation of the supernatant has been successfully used to purify the comparatively thermal-resistant VHHs (Olichon et al. 2007). Although periplasmic extraction has its benefits in terms of protein folding, it also has limitations, such as aggregation and low yields of proteins. The latter could be due to a number of reasons, including the secretion system saturation, the absence of adequate chaperone machinery that can inhibit improper folding at high expression rates, high proteolytic activity, and a lengthy-expression technique (Pleiner et al. 2015).

### Advantages of nanobodies

In terms of size, the single variable segment of the heavy chain antibodies is the smallest functional antigen-binding domain natively created by the adaptive immune system (Muyldermans 2013). The myriad uses of Nbs can be attributed to their exceptionally small and structurally convenient nature that in turn accounts for their fast tissue penetration and short half-life. In terms of antigen-binding capabilities, the diversity in the VHHs and VNAR loop structures dramatically expands the repertoire of the antigen-binding sites. This diversity also significantly affect their access to and interaction with more antigen’s clefts and buried epitopes, known as cryptic antigenic regions which are not usually accessible by conventional antibodies (Desmyter et al. 1996; Stanfield et al. 2004; Stijlemans et al. 2004). Another remarkable feature of Nbs’ antigen-binding paratopes is their ability to adopt flat, concave and convex configurations which easily favors their use against folded proteins and recessed epitopes (Chaikuad et al. 2014; Custódio et al. 2020; De Genst et al. 2006; Henry and MacKenzie 2018; Muyldermans and Smider 2016). Furthermore, it is assumed that sdAbs can access holed sites on membrane proteins including ion channels and G protein-coupled receptors (Henry and MacKenzie 2018; Wei et al. 2011).

With regard to their autonomous behavior, Nbs serve as effective building blocks for multi-domain compositions, such as bivalent or multivalent to improve affinity, or bispecific to cross-link independent antigens (Muyldermans 2021a). Since VHHs are monomeric in nature, they do not cluster in multimers like scFv molecules. Furthermore, considering their high solubility and stability, Nbs can be easily fused to each other without the mispairing and solubility challenges that face the scFv dimers and multimers (Bannas et al. 2017). Moreover, varying the valency of the Nb domains that target tumors can strengthen the cell-killing and downregulation effect on certain tumor cells (Bannas et al. 2017; Oliveira et al. 2010; Sadeghnezhad et al. 2019). To achieve this, linkers can be used to create multivalent or multispecific configurations of Nbs. Also, fusion with albumin or short peptide tags can be used to extend the half-life or facilitate their purification and detection (Bannas et al. 2017; Beirnaert et al. 2017; Zupancic et al. 2021a, b). Moreover, Nbs have been successfully fused with larger proteins called megabodies. The subsequent binding of these megabodies to smaller proteins, guided by the Nb specificity, could convert them into larger protein complexes. This allows their structural analysis by cryo-electron microscopy, which is otherwise not the best strategy for solving the structure of low-molecular weight proteins (Masiulis et al. 2019).

From the crystallization ability perspective, Nbs are easy to crystallize due to their small size. They also have several properties that aid in the crystallization of harsh proteins including;(i) the ability to block domain movement, (ii) the ability to hide mobile polysaccharides bounded proteins, and (iii) the ability to insert in clefts or between interfaces. Those properties stabilize loops or large complexes and assist in the solubilization of proteins with limited solubility or even provide beneficial crystal contacts for membrane proteins (Desmyter et al. 2015). Practically, Nbs significantly helped in the stabilization of G protein-coupled receptors in their active-state conformations (Steyaert and Kobilka 2011).

Finally, an attractive advantage of Nbs is their ability to cross the blood–brain barrier (BBB), unlike regular antibodies. This makes them unique potential diagnostic and therapeutic tools for the central nervous system (Li et al. 2012). Nbs are also showing potential as screening tools via genetic modifications that links them to fluorescent proteins and thus could be used as biosensors or to trace target antigens intracellularly in living cells (Rothbauer et al. 2006). They also present a detailed depiction of immune specificity in-display libraries and are easily adaptable to high-throughput screening (Gonzalez-Sapienza et al. 2017; Rahbarizadeh et al. 2011). Being an efficient diagnostic tool, Nbs became among the best tracers for non-invasive imaging for either positron emission tomography/computerized tomography (PET/CT) or single-photon emission computerized tomography (SPECT) imaging.

Since the original patent on Nbs expired in 2013 for Europe and 2017 for the US (Arbabi-Ghahroudi 2017), the biotechnological, academic, industrial, and therapeutics communities have been pushing to commercialize Nbs. Currently, the number of studies on unique and inventive compositions and applications of Nbs is rapidly increasing.

### Applications of Nbs in infectious diseases

Among the broad spectrum of applications in which Nbs have been successfully involved, the infectious disease domain comes with a big and impactful share. The developed Nbs for this purpose could be categorized into three main groups: therapeutic/prophylactic, diagnostic, and functional and structural elucidation tools.

#### Nbs as therapeutic and/or prophylactic tools in infectious diseases.

This is the largest category in which multiple Nbs have been tested and evaluated against different types of pathogens. For example, many Nbs have been tested to act as neutralizing agents for viral infections including foot & mouth disease virus (FMDV) (Harmsen et al. 2008), human immunodeficiency virus (HIV-1) (Forsman et al. 2008; Lutje Hulsik et al. 2013; McCoy et al. 2012), influenza A virus (Wei et al. 2011), the Middle-East respiratory syndrome coronavirus (MERS-CoV) (Wrapp et al. 2020), poliovirus (Strauss et al. 2016), rabies virus (Terryn et al. 2016), respiratory syncytial virus (RSV) (Rossey et al. 2017), rotavirus (Maffey et al. 2016), and lately the Covid-19 causing virus (SARS-CoV-2) (Chen et al. 2021; Schoof et al. 2020; Yang et al. 2023).

Among the strategies through which Nbs were used to treat infections is to target key moieties within the pathogen to block its pathogenesis. For example, to interfere with the ability of *Campylobacter* to colonize the host, Nbs were tested by targeting an outer membrane protein and the flagella (Vanmarsenille et al. 2018). Other Nbs targeted the F4 fimbriae of *E. coli* (Harmsen et al. 2005), the *Salmonella enterica* FilC flagellin (Huen et al. 2019), and the *Streptococcus mutans* adhesin (Krüger et al. 2006). Another group of Nbs were developed to target the toxins produced by some pathogens so that they block their toxic effects on the host’s cells. This category included Nbs against the *Bacillus anthracis* toxin (Shali et al. 2018), the *Clostridium botulinum* neurotoxin (Dong et al. 2010; Mukherjee et al. 2012), the *C. difficile* toxin (Hussack et al. 2018), the *E. coli* heat-labile toxin (Harmsen et al. 2009a, b) and the *Staphylococcus aureus* Toxic-Shock Syndrome Toxin (TSST-1) (Adams et al. 2009). Moreover, Nbs were generated to target other virulence factors such as the type III secretion system of *Pseudomonas aeruginosa* accordingly blocking the transfer of toxins to the host’s cell (De Tavernier et al. 2016), the urease enzyme of *Helicobacter pylori* inhibiting this key enzyme for the survival of the pathogen within the host (Fouladi et al. 2019), and the internalin B (InlB) of *Listeria monocytogenes* blocking bacterial invasion (King et al. 2018).

In viral pathogens, Nbs targeted surface structures to block entry to the host cell such as the Ebola envelope glycoprotein (Liu et al. 2017), the hepatitis B virus envelope protein S (Serruys et al. 2009), the hepatitis C E2 glycoprotein (Tarr et al. 2013), and others. Also for the viral pathogens, Nbs targeted viral replication as in the case of the Ebola nucleoprotein (Darling et al. 2017), the HCV RNA-dependent RNA polymerase (NS5B) (Thueng-in et al. 2012), and the nucleoproteins of the influenza A (Hanke et al. 2016) and the Marburg virus (Darling et al. 2017).

Among the therapeutic applications of Nbs in infectious diseases is their use for targeted drug delivery as has been demonstrated against Herpes simplex virus 2, where Nbs against glycoprotein D conjugated to the cytotoxic domain of the *P. aeruginosa* exotoxin acted as immunotoxins and were very effective in killing the virus-infected cells (Geoghegan et al. 2015). Also, Nbs directed against β-lactamases such as TEM-1 and BclI successfully inhibited the enzymatic activity of these enzymes and rendered the resistant pathogen susceptible to β-lactam antibiotics (Conrath et al. 2001).

#### Nbs as diagnostic tools in infectious diseases.

Another area in which Nbs are used actively is in the diagnosis of infectious diseases. Many Nbs targeted against moieties in the pathogens have been considered for diagnostics purposes. For example, the type 2 NS1 protein of the Dengue virus (Fatima et al. 2014), ORF2 of the hepatitis E virus (Arce et al. 2023), HIV capsid proteins (Helma et al. 2012), and other viral targets. In addition, Nbs are also used for the diagnosis of bacterial pathogens including *Acinetobacter baumannii* (Rasoulinejad and Gargari 2016), *Brucella* spp. (Abbady et al. 2011), *E. coli* (Salhi et al. 2020), *S. aureus* (Hu et al. 2021), and *Vibrio cholerae* (Goldman et al. 2006).

#### Nbs as structural and functional elucidation tools in infectious diseases.

Another application of Nbs in infectious diseases is the use as tools to elucidate the crystal structure of a pathogen-related protein or investigate its function. To this end, multiple Nbs have been developed. For instance, Nbs against the gp120 of HIV-1 were used to elucidate both its function and structure (Chen et al. 2010), while the function of the Nef protein of the same virus was studied using another Nb (Bouchet et al. 2011). In the case of bacterial pathogens, Nbs were used for structural biology studies of the MazEF toxin/antitoxin of *E. coli* (Lah et al. 2003), and that of the EpsJ pseudopillin of *V. vulnificus* (Lam et al. 2009).

An updated comprehensive list of the diverse applications of Nbs against viral and bacterial infectious diseases is presented in Tables 1 and 2, respectively.

**Table 1:** Nanobodies directed against viral antigens

Virus	Target	Potential application of produced Nb	References
African Swine Fever Virus (ASFV)	ASFV p30	Immunodetection	Zhao et al. (2023)
Classical Swine Fever Virus (CSFV)	CSFV E2	Immunodetection	Cao et al. (2023)
Dengue Virus	Type 2 NS1 protein	Immunodetection	Fatima et al. (2014)
Ebola	Envelope glycoprotein (GP)	Potential therapeutic & diagnostic purposes	Liu et al. (2017)
	A nucleoprotein (NP)	Replication inhibitor & diagnostic purposes	Darling et al. (2017) and Sherwood and Hayhurst (2013)
Foot & mouth disease virus (FMDV)	O1 Manisa strain antigens	Viral neutralization	Harmsen et al. (2007, 2008, 2009a, b)
	A peptide representing GH-loop of viral protein 1	Viral neutralization	Harmsen et al. (2007)
Hepatitis B virus (HBV)	Envelope protein (S) Core antigen of HBV	Therapeutic intrabodies	Serruys et al. (2009, 2010)
Hepatitis C virus (HCV)	NS3 protease	Biological probe for in vitro & in vivo studies	Martin et al. (1997)
	RNA Dependent RNA Polymerase (NS5B)	NS5B inhibitor	Thueng-in et al. (2012)
	E2 glycoprotein	Neutralization & inhibition of the HCV transmission	Tarr et al. (2013)
	C-terminal NS3 protein of HCV genotype 3a	HCV helicase inhibitor	Phalaphol et al. (2013)
	Recombinant NS3 & NS4A Fusion protease protein	HCV protease & replication inhibitor	Jittavisutthikul et al. (2015)
Hepatitis E virus (HEV)	HEV ORF2	Immunodetection	Arce et al. (2023)
Herpes Simplex Virus 2	Viral surface glycoprotein D	Delivery of specific effector molecules to infected cells	Geoghegan et al. (2015)
Human Immunodeficiency Virus (HIV-1)	Conserved envelope structures	Potent & broadly cross-reactive HIV-1 inhibitors	Chen et al. (2008)
	Envelope protein gp120	HIV-1 neutralization and/or vaccine candidate	Forsman et al. (2008), Hinz et al. (2010), Koh et al. (2010), Matz et al. (2013), and McCoy et al. (2012)
	Conserved structure on the viral envelope glycoprotein	Tools to study gp120 structures & understand mechanisms of entry	Chen et al. (2010)
	HIV-Rev protein	Rev-inhibitor blocking multimerization	Boons et al. (2014) and Vercruysse et al. (2010)
	Chemokine receptor CXCR4	CXCR4 blockers	Jähnichen et al. (2010) and Van Hout et al. (2018)
	HIV-1 Nef protein	Nef-inhibitor & a potential tool for studying Nef functions	Bouchet et al. (2011)
	Gp41 MPER-specific	HIV-1 neutralization	Lutje Hulsik et al. (2013)
	Viral protein R (Vpr) and capsid (CA)	Vpr cellular localization inhibitor & a potential tool for functional studies of Vpr	Matz et al. (2014)
	Coreceptor-binding site of gp120	Generate HIV-resistant cells	Jin et al. (2021)
	The capsid proteins	Blocking and/or diagnostic purposes	Alfadhli et al. (2021), Gray et al. (2017), Helma et al. (2012), Pezeshkian et al. (2019), and Tang et al. (2016)
Influenza A	M2 Ion Channel Protein	M2 inhibitor & virus neutralization	Wei et al. (2011)
	(N1–N9) Neuraminidase (NA)	Immunoaffinity purification of NA for industry Biosensors for virus detection ELISA reagent	Harmsen et al. (2013)
	Nucleoprotein (NP)	Virus replication inhibitor and/or crystal structure elucidation	Ashour et al. (2015), Hanke et al. (2016), and Schmidt et al. (2016)
H5N1 Influenza	Receptor binding site of HA 5	Viral neutralization	(Hultberg et al. 2011)
	Antigenic site B in HA 5	Viral neutralization	Ibañez et al. (2011)
	Hemagglutinin (HA)	Viral neutralization	Tillib et al. (2013)
	Neuraminidase (NA)	NA & viral replication inhibitor	Cardoso et al. (2014)
Marburg virus	Nucleoprotein	Viral replication inhibitor and/or diagnostic purposes	Darling et al. (2017) and Sherwood et al. (2007)
MERS-CoV	S protein	Viral neutralization	Wrapp et al. (2020)
Norovirus	Lower region of the protruding domain	Diagnostic & therapeutic potential	Koromyslova and Hansman (2015) and Salmen et al. (2023)
	Norovirus capsid	Inhibiting multiple stages of the virus life cycle	Koromyslova and Hansman (2017)
Poliovirus	Poliovirus type 1	Replication inhibitor	Thys et al. (2010)
	Capsid protein	Virus neutralization & standard for quality control	Strauss et al. (2016)
Porcine parvovirus	VP2 protein	Diagnostic purpose	Lu et al. (2020)
Porcine reproductive & respiratory syndrome virus	Viral non-structural protein 4	Replication inhibitor	Liu et al. (2016)
	Viral non-structural protein 9		Liu et al. (2015), and Wang et al. (2019)
	Glycoprotein 5		Liu et al. (2020)
	Nucleocapsid (N) protein (PRRSV-N-Nb1 &-Nb2)		Duan et al. (2023)
Rabies	Glycoprotein	Viral neutralization for prophylaxis	Hultberg et al. (2011) and Terryn et al. (2016)
Respiratory syncytial virus (RSV)	Fusion protein (F)	Viral neutralization	Hultberg et al. (2011) Detalle et al. (2015), and Rossey et al. (2017)
Rotavirus	Porcine retrovirus matrix domain protein p15	Inhibiting retrovirus production (very useful for xenotransplantation infections)	Dekker et al. (2003)
	Rhesus-monkey rotavirus serotype G3, strain RRV	Preventing or treating RV induced diarrhea	Martín et al. (2011), Pant et al. (2006), van der Vaart et al. (2006)
	Inner capsid protein VP6 of Group A rotavirus	Viral neutralization (diagnostic therapeutic and/or prophylactic purposes)	Garaicoechea et al. (2008), Gómez-Sebastián et al. (2012), Maffey et al. (2016), and Vega et al. (2013)
SARS-CoV-1	Receptor‐binding domain	Potent neutralizing activity	Gai et al. (2021)
	S protein	Potent neutralizing activity	Wrapp et al. (2020)
SARS-CoV-2	Receptor binding domain binding (RBD) of the virus spike protein	Viral neutralization for therapeutic, prophylactic, and/or diagnostic purposes	Chen et al. (2021), Custódio et al. (2020), Gai et al. (2021), Hanke et al. (2020), Huo et al. (2021), Koenig et al. (2021), Li et al. (2021), Nambulli et al. (2021), Pymm et al. (2021), Schoof et al. (2020), Walter et al. (2020), Wrapp et al. (2020), Wu et al. (2020), Xiang et al. (2020), Yang et al. (2023), Ye et al. (2020), and Zupancic et al. (2021a)
Tulip virus X	Whole virus particles	Immunological detection	Beekwilder et al. (2007)
Vaccinia	Western Reserve strain	Immune biosensor Immunoassay reagent	Goldman et al. (2006)
Vesicular stomatitis virus	Viral nucleocapsid N	Antiviral reagents	Schmidt et al. (2016)

**Table 2:** Nanobodies directed against bacterial antigens

Bacteria	Target	Potential application of produced Nb	References
*Acinetobacter baumannii*	Biofilm associated protein (Bap)	Immunoassay	Rasoulinejad and Gargari (2016)
*Bacillus anthracis*	Protective antigen (PA) toxin	Neutralization	Shali et al. (2018)
β-lactam resistant pathogens	βeta-Lactamase (TEM-1 & BcII)	β-lactamases inhibitor	Conrath et al. (2001)
*Brucella abortus*	Strain NalR	Therapeutic, prophylactic, and/or diagnostic purposes	Abbady et al. (2011)
*Brucella melitensis*	Strain Riv.1	Therapeutic, prophylactic, and/or diagnostic purposes	Abbady et al. (2011)
*Campylobacter*	Flagella	Reducing colonization	Riazi et al. (2013)
	Major outer membrane protein (MOMP)	Reducing colonization	Vanmarsenille et al. (2017)
	MOMP & flagella	Immunoprophylactic	Vanmarsenille et al. (2018)
*Clostridium botulinum*	Botulinum neurotoxin	Neurotoxin neutralization and/or diagnostic purpose	Conway et al. (2010), Dong et al. (2010), Goldman et al. (2008), Mukherjee et al. (2012), Thanongsaksrikul et al. (2010), and Tremblay et al. (2010)
*Clostridium difficile*	TcdA & TcdB toxins	Neutralization	Andersen et al. (2016), Hussack et al. (2018), and Yang et al. (2014)
*Clostridium tetani*	Tetanus toxoid & lysozyme	Nb functional studies	Arbabi Ghahroudi et al. (1997)
*Escherichia coli*	F4 fimbriae	Immunotherapeutic Inhibiting adhesion to intestinal brush	Harmsen et al. (2005)
	Surface antigens	Diagnostic & therapeutic purposes	Salhi et al. (2020)
	Heat-labile toxin	Toxin neutralization	Harmsen et al. (2009a, b)
	MazEF toxin/antitoxin system	Structural biology & crystallography	Lah et al. (2003)
*Helicobacter pylori*	Urease	Enzyme inhibition	Fouladi et al. (2019)
*Listeria monocytogenes*	Internalin B (InlB)	Prevention of bacterial invasion	King et al. (2018)
*Neisseria meningitidis*	Lipopolysaccharide	Therapeutic purpose against sepsis	El Khattabi et al. (2006)
*Pseudomonas aeruginosa*	PcrV of Type III secretion system T3SS	Blocking host cytotoxicity	De Tavernier et al. (2016)
*Salmonella enterica*	FliC Flagellin	Therapeutic purposes	Huen et al. (2019)
*Staphylococcus aureus*	Toxic-Shock Syndrome toxin-1(TSST-1)	Toxin neutralization	Adams et al. (2009)
	Enterotoxin B	Immunoassay	Hu et al. 2021 and Sun et al. (2020)
*Streptococcus mutans*	Streptococcal antigen I/II adhesin	Prophylaxis against dental caries	Krüger et al. (2006)
*Vibrio cholerae*	Cholera toxin	Immunoassay	Goldman et al. (2006)
*Vibrio vulnificus*	EpsJ pseudopilin	Structural biology & crystallography	Lam et al. (2009)

The majority of the reported Nbs that are listed in Table 1 are directed towards viral targets, which could be attributed to the Nbs advantages discussed earlier, especially their high accessibility and penetration capabilities. With the global concerns associated with the SARS-CoV-2 pandemic in the previous three years, there was a plethora of attempts to face this threat using Nbs platforms. Over fifteen studies, targeted engineered Nbs showed promising results in neutralizing the SARS-CoV-2 virus and suppressing mutational escape in different pre-clinical animal models. In addition, several Nbs studies have contributed to the ongoing efforts to find a cure for HIV infections using multiple approaches.

Compared to viral antigens, the application of Nbs in dealing with bacterial pathogens is still limited (Table 2). Up to date, the applications of using multiple Nbs have been directed to the neutralization of the botulinum neurotoxin. It is an attractive target for the development of monospecific antibodies owing to its extreme lethality and having the least LD_50_ value among known toxins. It is worth mentioning that the only currently FDA-approved treatment for botulism is an equine-driven polyclonal antibody cocktail shot (Tomic et al. 2021). Additionally, *E. coli* with its diverse pathogenic potentials attracted attention for the development of therapeutic Nbs either for blocking attachment (Harmsen et al. 2005) or toxin neutralization (Harmsen et al. 2009a, b).

On another front, very few attempts have been implemented in the production of anti-fungal Nbs. Most of the studies aimed at detecting food product contamination with mycotoxins, specifically the aflatoxin B_1_ (He et al. 2022; Salvador et al. 2022). Recently, Liu et al (2023) designated Nb-natamycin conjugates that were specific to the *Aspergillus fumigatus* β-glucan. *A. fumigatus* is known to be a common causative agent of fungal keratitis, an inflammatory eye disease affecting the cornea. These conjugates successfully attenuated the virulence of *A. fumigatus,* and favorably modulated the inflammatory responses in fungal keratitis (Liu et al. 2023). Earlier, the same group described another Nb that is specific to the mammalian pattern-recognition receptor for fungi dectin 1. The anti-dectin 1 Nb alleviated the clinical symptoms of fungal keratitis in a mouse model, and this was attributed to the reduced expression of inflammatory cytokines IL-1β and IL-6 (Liu et al. 2022).

### Disadvantages of nanobodies

The Nbs technology has become successively incorporated in a lot of therapeutic and diagnostic applications due to its small size. However, Nb's small size accounts for its short half-life by being rapidly eliminated by kidneys. This is attributed to their low molecular weight (~ 15 kDa) which is below the renal threshold for glomerular filtration (~ 50 kDa) (Ruggiero et al. 2010). Hence, their diminutive size and thereby their short half-life accounts for some challenges or limitations for using Nbs in different therapeutic fields such as screening and in vivo diagnosis applications. One of these challenges is the high uptake and accumulation of Nbs in the kidneys while being eliminated, which in turn limits their use as in vivo imaging probes for kidney screening along with some vicinity organs like the pancreas (Schoonooghe et al. 2012). In addition, the binding capacity of some Nbs is altered after being conjugated with either fluorophore or radioactive probes, for example, the Nbs conjugated with chelators having gallium-68 (^68^Ga) or zirconium-89 (^89^Zr) for immuno-positron emission tomography (immunoPET) imaging. These radiolabeled nanobodies may exhibit different features, including affinity, size, structure, and pharmacokinetics. However, site-directed conjugation and nanobody-engineering strategies have been recently applied to demonstrate the effectiveness, reliability, and safety of their use as molecular imaging probes (Yang et al. 2022). Another challenge is the low persistence of Nbs within the bloodstream due to their rapid clearance which in turn hampers their uptake. As a result, only a negligible fraction of the administered nanobody reaches the target sites, thereby hindering their efficacy. This may account for the frequent administration of Nbs along with using higher doses to maintain their therapeutic level, however, this is not recommended for an efficient therapeutic application. Further approaches seek different strategies to prolong Nbs half-life by enhancing their accumulation and pharmacokinetics either by Nbs multimerization approach or by Nb-serum albumin conjugation approach (Jovčevska and Muyldermans 2020).

Lastly, due to the high homology between the camelid germline IgV gene repertoire and its human counterparts, with up to 95% in the case of the camelid IGHV family III and its human FR counterpart, Nbs inherently pose a low immunogenic profile, which allows for prolonged and repeated administrations of Nbs in patients (Klarenbeek et al. 2015). However, the generation of antibodies against administered Nbs is possible and can be problematic, as demonstrated in the aborted clinical trial with an anti-DR5 receptor nanobody (Papadopoulos et al. 2015). This suggests that moderate humanization of Nbs sequences may be beneficial in some cases.

Despite of these challenges and limitations, the Nb technology still shows significant advantages over the conventional antibody as an effective immunotherapy.

## Conclusions

Nbs represent a very promising tool for a vast array of biomedical applications owing to their superiority in terms of small molecular size, modulative specificity, and their physico-chemical properties that allow for easier downstream processing. The majority of current Nb applications are focused on the fields of diagnostics, and structural biology, being used as structural aids for troublesome proteins. The recent surge in anti-viral development accelerated the expansion in therapeutic Nb research, with many promising candidates designed to target viral infections. Anti-bacterial and anti-fungal Nb candidates are still limited in numbers and targets, which calls for future investigation of their potential applications for this purpose. This could be especially warranted in the post-antibiotic era, where available antibiotics are failing to suppress extremely resistant microbes.

## Data Availability

The datasets generated during and/or analyzed during the current study are available from the corresponding author on reasonable request.
